# Familial risks in nervous system tumours: joint Nordic study

**DOI:** 10.1038/sj.bjc.6605708

**Published:** 2010-05-25

**Authors:** K Hemminki, S Tretli, J H Olsen, L Tryggvadottir, E Pukkala, J Sundquist, C Granström

**Affiliations:** 1 Division of Molecular Genetic Epidemiology, German Cancer Research Center (DKFZ), Heidelberg, Germany; 2 Center for Family and Community Medicine, Karolinska Institute, Huddinge, Sweden; 3 Norwegian Cancer Registry, Oslo, Norway; 4 Institute of Cancer Epidemiology, Copenhagen, Denmark; 5 Icelandic Cancer Registry, Reykjavik, Iceland; 6 Department of Medicine, University of Iceland, Reykjavik, Iceland; 7 Finnish Cancer Registry, Institute for Statistical and Epidemiological Cancer Research, Helsinki, Finland; 8 School of Public Health, University of Tampere, Tampere, Finland; 9 Center for Primary Care Research, Lund University, Malmö, Sweden; 10 Stanford Prevention Research Center, Stanford University School of Medicine, Palo Alto, CA, USA

**Keywords:** brain tumour, spinal tumour, peripheral nerve tumour, familial risk

## Abstract

**Background:**

Familial nervous system cancers are rare and limited data on familial aspects are available particularly on site-specific tumours.

**Methods:**

Data from five Nordic countries were used to analyse familial risks of nervous system tumours. Standardised incidence ratios (SIRs) were calculated for offspring of affected relatives compared with offspring of non-affected relatives.

**Results:**

The total number of patients with nervous system tumour was 63 307, of whom 32 347 belonged to the offspring generation. Of 851 familial patients (2.6%) in the offspring generation, 42 (4.7%) belonged to the families of a parent and at least two siblings affected. The SIR of brain tumours was 1.7 in offspring of affected parents; it was 2.0 in siblings and 9.4 in families with a parent and sibling affected. For spinal tumours, the SIRs were much higher for offspring of early onset tumours, 14.0 for offspring of affected parents and 22.7 for siblings. The SIRs for peripheral nerve tumours were 16.3 in offspring of affected parents, 27.7 in siblings and 943.9 in multiplex families.

**Conclusion:**

The results of this population-based study on medically diagnosed tumours show site-, proband- and age-specific risks for familial tumours, with implications for clinical genetic counselling and identification of the underlying genes.

Some 90% of nervous system tumours are located in the brain whereas spinal and peripheral nerve tumours account for the remainder (Centre for Epidemiology, 2007). Recently large epidemiological studies on nervous system cancer have been carried out but a few environmental risk factors have consistently been identified (Bondy *et al*, 2008). Therapeutic and low-level irradiation, hereditary syndromes and family history remain as the established risk factors of nervous system tumours (Stewart and Kleihues, 2003; Hijiya *et al*, 2007; Maule *et al*, 2007; Hemminki *et al*, 2008a). Less than 3% of patients with nervous system tumours have a first-degree family member diagnosed by these neoplasms (Hemminki *et al*, 2008b). Because of the low incidence and rarity of familial cancers many of the published genetic epidemiological studies have not been able to distinguish anatomic locations or tumour types with sufficient numbers of cases (Goldgar *et al*, 1994; Hemminki *et al*, 1998, 2001a, 2009; Malmer *et al*, 1999, 2001, 2002; Paunu *et al*, 2002; Hemminki and Li, 2003; Blumenthal and Cannon-Albright, 2008). Although the data on familial risks in nervous system cancer are overwhelmingly positive showing an effect, the data from Iceland show no effects (Amundadottir *et al*, 2004). By contrast, the data on childhood brain tumours has not shown strong familial effects with some exceptions (Olsen *et al*, 1995; O’Neill *et al*, 2002; Searles Nielsen *et al*, 2008; Hemminki *et al*, 2009).

To overcome the problems of small numbers of familial nervous system tumours, we carried out a joint study in five Nordic countries all of which have population records to assemble families and nationwide cancer registries. Moreover, the incidence rates do not appreciably differ between the Nordic countries, all rates remaining in the interval 5.9–7.8 per 100 000 for men and 4.6–6.5 per 100 000 for women (IARC, 2002). Joint Nordic family studies have been carried out before but they have focused on childhood cancer because in many of the participating countries the family linkages were possible only between parents and relatively young offspring (Sankila *et al*, 1998; Olsen *et al*, 2001; Winther *et al*, 2001). In this landmark study we show that joint Nordic family studies are feasible by extending the second generation at least to mid age in all five countries and thus covering a population base of 25 million people. A histology-specific analysis was recently reported on the Swedish and Norwegian data sets (Hemminki *et al*, 2009). In this study also Danish, Finnish and Icelandic data sets were included and a novel analysis distinguished tumours in the brain, spinal cord and peripheral nerves. A total of 63 307 patients with nervous system cancers were identified and among them 851 in the offspring generation had a family history. In addition to being the largest study yet conducted, the other advantages include registered and compete family structures and complete medically diagnosed cancers from nationwide registries.

## Subjects and methods

The five Nordic countries have population registers through which any offspring with a nervous system tumour can be identified whose parents or siblings were also diagnosed with nervous system tumour. With the exception of Iceland, sibships could only be ascertained in the offspring generation.

Statistics Sweden maintains a Multigeneration Register that covers offspring born after 1931 along with their parents. We have linked this Register to the Swedish Cancer Registry (1958–2004) to make the Family-Cancer Database (MigMed2) in year 2006 with a total population of over 11.5 million individuals. In addition to the native Swedes the Family-Cancer Database contains data on all immigrants residing in Sweden. In the Database all data are organised in child–mother–father triplets; the parents have been registered at the time of birth of the child, allowing tracking of biological parents. The completeness of cancer registration in the 1970s has been estimated to be over 95%, and is now considered to be close to 100%. The percentage of cytologically or histologically verified cases has been close to 100%. The Swedish Cancer Registry is based on compulsory notification of cases. A four-digit diagnostic code according to the *International Classification of Diseases*, 7th revision according to the International Classification of Diseases for Oncology, World Health Organization (WHO) was combined with a three-digit pathological anatomic diagnosis (PAD) code provided by the Cancer Registry. Cranial nerve tumours were included among brain tumours. The Swedish data included 34 934 nervous system cancer patients. The follow-up was from 1961 to 2004.

The Norwegian data source covers all people born in Norway between 1900 and 2005, totalling 6.4 million individuals. Data on familial relationships, birth, vital status and sex were collected from the Central Population Register, which was linked to the national Cancer Registry of Norway. Offspring linkage to parents was almost complete for Norwegian-born offspring since 1954 and available for foreign-born offspring from 1960. Likewise, information on date of death and emigration was available from 1960.

The Norwegian data included 19 317 nervous system cancer patients. The follow-up was from 1953 to 2005.

The Danish proband population data, born between 1935 and 2003, and diagnosed with brain or spinal cord cancer between 1978 and 2003, were retrieved from the nationwide Danish Cancer Registry. Malignant and benign tumours were included. Peripheral nerve tumours, acoustic neurinomas and other cranial nerve tumours were excluded because of variable reporting. First-degree relatives were identified for probands by linkage to the Danish Central Population Register, which provides data on birth, sex, vital status and familial relationships in all the Danish population since 1 April 1968. A Danish modified version of the *International Classification of Diseases*, 7th revision according to the International Classification of Diseases for Oncology, WHO was used to classify the cancers. Danish age-, sex- and period-specific incidence rates of each type of cancer were used for person-years calculation with follow-up period 1967–2003. The Danish data included 7941 nervous system cancer patients.

The Icelandic Genealogy Database contains verified and updated information on the genealogies of all Icelanders dating back to birth year 1840. The database was constructed under the auspices of the Genetical Committee of the University of Iceland, starting in 1965. It was created by linking between the 1910 census and the Icelandic National Registry (founded in 1953), and then completed for all Icelanders born after 1839 by adding information on the period 1840–1910 from parish records and censuses. The database has been the basis for numerous scientific investigations, for more than three decades. From the Icelandic Cancer Registry, known to cover more than 99% of all cancer diagnosed in Iceland, probands of all ages and diagnosed with brain cancer between 1955 and 2005 were linked to the Genealogy Database to trace first-degree relatives. A patient was kept as a proband, although he or she was a relative in another family. Information was obtained on vital status and gender. The follow-up period started in January 1955 or at birth, whichever came first, and ended on 31 December 2005 or at death. The Icelandic data included 1120 nervous system cancer patients.

In Finland, the national population registration system at Population Register Center includes links to parent for offspring younger than 20 years in October 1973 when the links were first created; also older offspring were linked to parents if they lived in the parental address in 1973. Thus parents can be automatically traced virtually for every person born in October 1953 or later. Finnish Cancer Registry is known to cover practically all cancers diagnosed in Finland. The Finnish data included 1370 nervous system cancer patients. The follow-up was from 1953 to 2005.

Standardised incidence ratios (SIRs) were used to measure the cancer risks for offspring according to occurrence of cancers in their families. Standardised incidence ratios were calculated for offspring whose parent, sibling or parent and sibling had the same, concordant cancer, that is using parents or sibling as probands. Follow-up was started for each offspring at birth, immigration or 1 January, of the country-specific year, stated above, whichever came latest. Follow-up was terminated on diagnosis of first cancer, death, emigration or the closing date of the study, 31 December 2003/2004/2005, depending on the country. When more than two affected offspring were found in any family, they were counted as independent event. Standardised incidence ratios were calculated as the ratio of observed (O) to expected (E) number of cases. The expected numbers were calculated from 5-year-age-, sex-, tumour type- and period- (5-year bands) specific standardised incidence rates for all offspring lacking a family history (Esteve *et al*, 1994). Confidence intervals (95% CI) were calculated assuming a Poisson distribution (Esteve *et al*, 1994). Risks for siblings were calculated using the cohort method, described and discussed elsewhere (Hemminki *et al*, 2001b). In this method, sibships of two or more are included and all siblings contribute to cases and person years. Families with multiple affected individuals are ascertained at multiple times and they are not independent, leading to too narrow CIs (approximately by a factor of 1.4, Hemminki *et al*, 2001b); no correction was carried out in this article.

## Results

The joint Nordic study results are shown in Table 1. Among 32 347 offspring patients with nervous system cancer, 851 were familial cases, thus accounting for 2.6% of all patients. Considering additionally the parental probands, 1381 familial cases were identified. Familial risks were calculated when probands presented with any nervous system cancers. The SIRs were higher for siblings than for offspring of parental probands, and they were highest when both a parent and a sibling were probands (multiplex families, SIR 7.85). Affected offspring in multiplex families numbered 42, that is they accounted for 4.7% of the offspring with familial nervous system tumours. When cases were found in 0- to 19-year olds, the SIRs were higher than those in older cases.

In Table 2, site-specific familial risks are shown in two age groups (0–19 and 20+ years) and separately for a concordant family history (brain–brain, spine–spine etc.) and a family history of nervous system tumours. The Finnish data were not included in this and later analysis because of the small number of cases. Brain cancer accounted for 87.6% of all offspring cases and 93.7% of the familial cases, spinal tumours accounted for 6.7% of all and 1.3% of the familial cases, peripheral tumours accounted for 5.7% of all and 6.0% of the familial cases. The early onset cases were a minority of all cases at each anatomic site but among familial spinal and peripheral nerve patients they were the majority. For concordant anatomic sites, peripheral nerves showed the highest risk, followed by the spine and the brain. Young patients had higher risks than the older ones, and multiplex families (parent and sibling proband) showed higher risks than siblings or offspring of affected parents. The SIR of brain tumours was 1.7 in offspring of affected parents, 2.0 in siblings and 9.4 in multiplex families. For spinal tumours, the SIRs were much higher for early onset tumours, 14.0 for offspring of affected parents and 22.7 for siblings. The SIRs for peripheral nerve tumours were 16.3 in offspring of affected parents, 27.7 in siblings and 943.9 in multiplex families. When the probands were diagnosed with nervous system tumours, instead of those in concordant anatomic sites, the SIRs for brain tumours increased but those for the other sites decreased.

In Table 3, the SIRs for early and late onset tumours are considered for cases and probands by concordant anatomic site only. The early onset brain tumours were increased in offspring of affected parents (4.79) and in siblings (6.06). The highest risk of the study, SIR 2563, was observed for peripheral nerve tumours in a single multiplex family.

## Discussion

We aimed at testing the feasibility of familial linkages for adult cancers in all the Nordic countries, which would enable future collaboration with global visibility in a current base population of 25 million. All the countries have nationwide cancer registries started for more than half a century ago. Although with some national features, particularly for Iceland with a genealogical database, the basic linkage of family members was performed through the personal identifier on which the national population registers have assembled the family members. The age of the second generation depended on the period when the personal identifier was introduced, except for Iceland. It was introduced first in Sweden, in the year 1947, and those who were 15 years at that time, that is born in the year 1932, were linked to their parents; thus the highest possible offspring age was 72 years in Sweden. There was no age limit in Iceland whereas for the remaining Nordic countries, the second generation aged up to 50s. Nervous system cancer was selected for study because it is of relatively early onset and thus many cases were recorded even in the second generation.

In this analysis we wanted to examine familial clustering by using three types of probands: ‘parent only’, ‘sibling only’ and ‘parent and sibling’ (multiplex families) to deduce the possible inheritance and penetrance modes. Dominant and recessive effects could be distinguished by a higher risk for siblings than for offspring of affected parents. However, a similar effect would be caused by environmental sharing in childhood, which could be related to sibship size and order in a sibship as surrogates of infections causes (Altieri *et al*, 2006). Another reason for a higher risk among siblings than among offspring of affected parents could be mortality in young patients before parenthood. Even though survival in brain tumours has increased, over half of the patients have died in 5 years; however, the survival depends on the tumour type and it is better for benign tumours such as meningioma compared with glioma (Talback *et al*, 2003). For brain and spinal tumours, the excess sibling risk was found among early onset cases, which may suggest a higher role for genetic effect than for survival selection. Thus, even though for all the three anatomic sites sibling risks exceeded those for offspring of affected parents the data should be interpreted with caution. Multiplex families are, however, likely to be due to high or moderate penetrance genetic effects. In addition to the high penetrant genes causing syndromatic nervous system tumours (Kleihues and Cavenee, 2000), a recent study identified five low-penetrant loci for glioma with an estimated contribution of 7–14% to the familial clustering of glioma (Shete *et al*, 2009).

Familial cases accounted for 2.6% of all patients in the offspring generation, in line with previous estimates (Goldgar *et al*, 1994; Hemminki *et al*, 2008b). Affected offspring in multiplex families accounted for 4.7% of the offspring with familial nervous system tumours, which indicates that most familial clusters are non-syndromatic presenting with modest penetrance at most. Rare cancer syndromes such as Li-Fraumeni, neurofibromatosis 1 and 2, von Hippel–Lindau, tuberous sclerosis, Turcot and Gorlin would manifest in multiplex families (Kleihues and Cavenee, 2000; Vogelstein and Kinzler, 2002). The very high risks for peripheral nerve tumours in the multiplex families were probably due to neurofibromatosis 1 and 2, which are often of early onset as in this study. Gliomas also manifest in these syndromes and in Li-Fraumeni syndrome and tuberous sclerosis.

Most of the nervous system cancers were located in the brain (87.6%) and even larger percentage of concordant familial cases were found there (93.7%). However, the concordant familial risks were much higher for spinal and peripheral nerve tumours compared with brain tumours. These high risks could partially be explained by syndromatic cases in small families, discussed above. The study showed that there is a clear familial risk in early onset brain tumours both through parental and sibling probands, and also when probands were diagnosed before age 20 years.

The present population-level data on medically diagnosed cases from the five Nordic countries provide solid risk estimates for familial nervous system tumours. These data should be useful for the clinical setting by offering cancer patients and their relatives unbiased estimates on and objective advice about familial cancer (Hemminki and Eng, 2004). Second, these data should be useful in the planning of gene identification efforts before the projects have been initiated and in the estimation of their success after new susceptibility genes have been identified (Hemminki *et al*, 2006). To reach the ultimate goal of genetic understanding of nervous system tumour aetiology, we need to account the detected genes for all of the genetic familial aggregation of this disease.

## Figures and Tables

**Table 1:** Estimates of SIRs for familial risk of offspring nervous system tumours in the Nordic countries

	**Sweden, Norway, Denmark, Finland and Iceland**													
	**Parental proband**				**Sibling proband**				**Parent and sibling proband**					
**Age of cases**	**O**	**SIR**	**95% CI**		**O**	**SIR**	**95% CI**		**O**	**SIR**	**95% CI**		**No family history**	**All**
0–19 years	125	**2.24**	1.87	2.67	54	**2.71**	2.03	3.54	10	**23.47**	11.18	43.34	7401	7590
20+ years	384	**1.58**	1.42	1.74	246	**1.85**	1.63	2.10	32	**11.80**	8.06	16.67	24 095	24 757
All ages	509	**1.70**	1.56	1.86	300	**1.97**	1.75	2.20	42	**13.38**	9.64	18.10	31 496	32 347

Abbreviation: SIR=standardised incidence ratio.

SIR adjusted for age, sex and 5-year periods. Values given in bold indicate that 95% CI do not include 1.0.

**Table 2:** Estimates of SIRs for familial risk of early and late onset nervous system tumours in offspring

	**Sweden, Norway, Denmark and Iceland**																								
	**SIR by concordant family history**												**SIR by family history of cancer in the nervous system**												
	**Parental proband**				**Sibling proband**				**Parent and sibling proband**				**Parental proband**				**Sibling proband**				**Parent and sibling proband**				
**Subsite and age in cases**	**O**	**SIR**	**95% CI**		**O**	**SIR**	**95% CI**		**O**	**SIR**	**95% CI**		**O**	**SIR**	**95% CI**		**O**	**SIR**	**95% CI**		**O**	**SIR**	**95% CI**		**Cases**
*Brain*																									
0–19 years	67	**1.7**	1.3	2.1	35	**2.7**	1.9	3.7	—	—			86	**1.9**	1.5	2.3	40	**2.6**	1.8	3.5	5	**15.3**	4.8	35.9	5751
20+ years	286	**1.7**	1.5	1.9	166	**1.9**	1.6	2.2	17	**10.8**	6.3	17.3	314	**1.6**	1.4	1.8	19.5	**1.9**	1.6	2.2	20	**9.5**	5.8	14.7	20 991
All ages	353	**1.7**	1.5	1.8	201	**2.0**	1.7	2.3	17	**9.4**	5.5	15.1	400	**1.7**	1.5	1.8	235	**2.0**	1.7	2.2	25	**10.3**	6.7	15.2	26 742
																									
*Spine*																									
0–19 years	3	**14.0**	2.6	41.4	2	**22.7**	2.1	83.7	—	—			14	**3.6**	2.0	6.1	4	3.0	0.8	7.6	—	—			440
20+ years	1	**1.4**	0.0	8.2	2	4.2	0.4	15.5	—	—			22	1.5	0.9	2.2	16	**2.1**	1.2	3.4	2	**12.8**	1.2	47.0	1590
All ages	4	**4.4**	1.1	11.3	4	**7.1**	1.8	18.4	—	—			36	**1.9**	1.3	2.6	20	**2.2**	1.4	3.4	2	**10.8**	1.0	39.9	2030
																									
*Peripheral nerves* a																									
0–19 years	11	**29.8**	14.8	53.5	5	**24.7**	7.8	58.1	—	—			15	**2.5**	1.4	4.1	8	**3.8**	1.6	7.6	3	**63.0**	11.9	186.6	685
20+ years	5	**8.2**	2.6	19.2	12	**29.2**	15.0	51.1	4	**1816.7**	472.5	4697.6	22	1.5	0.9	2.3	19	**2.6**	1.6	4.1	7	**45.5**	18.0	94.3	1037
All ages	16	**16.3**	9.3	26.6	17	**27.7**	16.1	44.4	4	**943.9**	245.5	2440.6	37	**1.8**	1.3	2.5	27	**2.9**	1.9	4.2	10	**49.7**	23.6	91.7	1722
																									
*Nervous system*																									
0–19 years													118	**2.1**	1.8	2.6	54	**2.9**	2.2	3.8	8	**20.4**	8.7	40.5	6963
20+ years													375	**1.6**	1.4	1.7	240	**1.9**	1.6	2.1	29	**11.1**	7.4	16.0	24 374
All ages													493	**1.7**	1.5	1.8	294	**2.0**	1.8	2.2	37	**12.3**	8.7	17.0	31 337

Abbreviation: SIR=standardised incidence ratio.

SIR adjusted for age, sex and 5-year periods.

a Denmark not included. Values given in bold indicate that 95% CI do not include 1.0.

**Table 3:** SIR for familial early and late onset nervous system tumours according to proband age groups

	**Sweden, Norway, Denmark and Iceland**																							
	**SIR by concordant family history**																							
	**Parent 0–19 years**				**Parent 20+ years**				**Sibling 0–19 years**				**Sibling 20+ years**				**Parent and sibling 0–19 years**				**Parent and sibling 20+ years**			
**Subsite and age in cases**	**O**	**SIR**	**95% CI**		**O**	**SIR**	**95% CI**		**O**	**SIR**	**95% CI**		**O**	**SIR**	**95% CI**		**O**	**SIR**	**95% CI**		**O**	**SIR**	**95% CI**	
*Brain*																								
0–19 years	4	**4.8**	1.2	12.4	63	**1.6**	1.2	2.1	35	**6.1**	4.2	8.4	—	—			—	—			—	—		
20+ years	—	—			286	**1.7**	1.5	1.9	8	1.2	0.5	2.4	158	**1.9**	1.7	2.3	—	—			17	**11.7**	6.8	18.8
All ages	4	**4.0**	1.0	10.2	349	**1.6**	1.5	1.8	43	**3.5**	2.5	4.7	158	**1.8**	1.5	2.1	—	—			17	**10.5**	6.1	16.8
																								
*Spine*																								
0–19 years	—	—			3	**14.4**	2.7	42.7	2	**54.0**	5.1	198.5	—	—			—	—			—	—		
20+ years	—	—			1	1.4	0.0	8.2	—	—			2	4.6	0.4	16.8	—	—			—	—		
All ages	—	—			4	**4.4**	1.1	11.4	2	**27.1**	2.6	99.7	2	4.1	0.4	15.0	—	—			—	—		
																								
*Peripheral nerves* a																								
0–19 years	—	—			11	**32.5**	16.1	58.3	5	**47.3**	14.9	111.3	—	—			—	—			—	—		
20+ years	1	182.1	0.1	1044.0	4	**6.6**	1.7	17.1	2	**24.7**	2.3	90.8	10	**30.3**	14.4	55.9	2	**2563.0**	241.6	9425.8	2	**1407.0**	132.6	5174.5
All ages	1	28.2	0.0	161.9	15	**15.9**	8.9	26.3	7	**37.5**	14.9	77.7	10	**23.4**	11.1	43.2	2	**917.3**	86.5	3373.4	2	**972.1**	91.6	3574.9

Abbreviation: SIR=standardised incidence ratio.

SIR adjusted for age, sex and 5-year periods.

a Denmark not included. Values given in bold indicate that 95% CI do not include 1.0.
